# Technology-facilitated Sexual Violence in South Korea: A Content Analysis of a Website for Victims

**DOI:** 10.1177/10778012231172712

**Published:** 2023-05-10

**Authors:** Soojeong Kim, Eunju Choi, Jane Dimmitt Champion

**Affiliations:** 1 School of Nursing, The University of Texas at Austin, Austin, TX, USA; 2 University of Texas MD Anderson Cancer Center, Houston, TX, USA; 3 School of Nursing, The University of Texas at Austin, Austin, TX, USA

**Keywords:** Technology-facilitated sexual violence, South Korea, qualitative research, mental health

## Abstract

Technology-facilitated sexual violence (TFSV) is an emerging form of gender-based violence. An understanding regarding the circumstance of TFSV and related health outcomes is limited. This qualitative study analyzed postings from an anonymous Korean website on which women suffering from TFSV freely posted messages asking for help and voicing concerns. Victims made efforts to solve problems that remained unresolved, thereby creating difficulties in their daily lives, and compelling them to quit jobs or break social relationships. They reported mental health concerns including suicide ideation, anxiety, frustration, sleep disorders, and depression. Results infer the imperative nature of research, intervention, and political action.

## Introduction

Sexual violence is defined as

Any sexual act, attempt to obtain a sexual act, unwanted sexual comments or advances, or acts to traffic, or otherwise directed, against a person's sexuality using coercion, by any person regardless of their relationship to the victim, in any setting, including but not limited to home and work. (World Health Organization, 2012, p. 2)

The term sexual violence refers not only to physical injury but also to psychological, social, and structural problems (Powell & Henry, 2017). With the development of information technology devices such as smartphones and tablets with which people access the Internet every day, the sharing of various sexually explicit photographs, messages, and videos is more common, and has an addictive aspect (Huh, 2017). Digital technology can also be used as a tool to facilitate sexual-based damage as Internet-enabled devices are rapidly spreading online communication services. According to a survey of 4,248 US adults conducted by the National Pew Research Center, 41% of US adults have been bullied online personally, and 66% have witnessed bullying (Duggan, 2017).

Despite the growing dominance of sexual violence using digital technology, a consistent, conceptual, and operational definition of the current phenomenon is lacking (Snaychuk & O’Neill, 2020). Various terms used in previous studies to describe this recently identified form of sexual violence include, revenge pornography (Hall & Hearn, 2017), cybervictimization (Reyns et al., 2012), electronic aggression (Bennett et al., 2011), and electronic harassment (Fenaughty & Harré, 2013). Recently, the term technology-facilitated sexual violence (TFSV) has emerged to bridge the gaps among the various terms. According to Powell and Henry (2019), TFSV is defined as a broad scope of digital sexual harassment that includes online sexual harassment, gender- and sexuality-based harassment, cyberstalking, image-based sexual exploitation, and coercion of a victim into an unwanted sexual act in the online space. Even though there are inevitable redundancies between different dimensions, Henry and Powell (2018) provided a further explanation of each dimension. Online sexual harassment, the first dimension, is defined as unwanted sexual attention in an online space or communications of sexual desires toward another. Second, gender and sexuality-based harassment refers to a process of harm and distress, including gender and sexuality-based hate speech, reputation-damaging lies, rape threats, false accusations of sexual violence, impersonation, and virtual rape. Cyberstalking is defined as an extension of offline stalking using electronic devices. Image-based sexual exploitation, also known as revenge pornography, is defined as the creation, distribution, or threat of distribution of intimate or sexually explicit images without a person's consent. Lastly, coercion of a victim into an unwanted sexual act in the online space is a form of blackmail, bribery, or intimidation, such as requiring the victim to engage in either online or in-person sex acts or demanding the release of intimate images or information.

The damage incurred by TFSV victimization is similar to sexual violence victimization. As with TFSV, sexual violence victimization is associated with adverse psychological effects such as feelings of hopelessness, depression, anxiety, post-traumatic stress disorder, and suicidal behaviors (Brake et al., 2019; Coles et al., 2015; Temple et al., 2007), TFSV victimization has broad, negative impacts on a person's well-being. Previous findings indicate that TFSV victimization among adult women was associated with the same detrimental mental health outcomes as sexual violence victimization (e.g., depression, anxiety, post-traumatic stress disorder, and suicidal ideation) (Bates, 2017; Cripps, 2016; Kamal & Newman, 2016). Champion et al. (2021) found that TFSV victimization increases suicide risk via a chain of bullying, depression, and perceived burdensomeness among adult men and women. Despite the similarities in outcomes reported for sexual violence and TFSV studies, victims of TFSV can be targeted anytime and anywhere, even within the physical safety of their homes, thereby creating a pervasive sense of threat (Snaychuk & O’Neill, 2020). For this reason, TFSV victims might experience adverse social and professional consequences, such as withdrawal from their workplaces or schools and difficulty remaining anonymous in communities where personal information was spread (Citron & Franks, 2014).

Korea ranked first in technology usage among 39 countries, with 94% of adults using smartphones as well as the Internet frequently regardless of education and age level (Poushter et al., 2018). Of women living in Seoul, Korea in 2019, 43% reported an experience of TFSV directly or indirectly (Seoul & Seoul Foundation of Women & Family, 2019). Direct TFSV refers to women who have experienced TFSV firsthand, and indirect TFSV refers to women who witnessed such violence and were impacted indirectly because of the idea that they might also be victims. These two types of victims are different; however, this survey did not separate them. According to a crime analysis in 2016 by Korean police, the most rapid increase in sexual assault crimes in the past decade involved “camera-based crimes,” which went from 3.6% in 2006 to 24.9% in 2015 (Kim & Chung, 2018). Due to rapid propagation in cyberspace, the permanent deletion of sexual photos or videos is impossible, and victims live with severe emotional pain. Moreover, as modern people use the Internet and social media to perform social functions and communicate with others, the damage of TFSV is considered to be a social killing that causes individuals to shrink socially, and compels them to live as if they were dead, as they are likely to disappear from their ordinary lives (Gwon, 2018).

The largest Korean pornographic website, “Soranet,” which included child pornography and videos from hidden cameras in public toilets, houses, and hotels, had more than 1 million members, before the government forced it to close in 2016. The closure of “Soranet” raised the issue of TFSV in Korean society (Huh, 2017). More than 200,000 Korean individuals requested reform to sexual assault law to strengthen the punishment of perpetrators, after a female celebrity died in 2019 by suicide, following threats to disseminate sexual videos obtained by her ex-boyfriend (Lee, 2019). Even though there was no physical violence, social media is the primary venue for TFSV, which can lead to death (Facts and figures, 2019). Ironically, according to interviews with the Korean Cyber Sexual Violence Response Center, it is difficult to ascertain individual situations. This is because victims do not want to reveal themselves and instead turn to artificial intelligence counselors, the Internet, or other social media to find help or information rather than human counselors, whom they would have to see face-to-face (Sowell, 2020). Consequentially, little is currently known about the prevalence, experiences, and impact of TFSV among victims in the Korean population. In addition, the victim's situations and the mental or social consequences of TFSV remain unclear (Reed et al., 2019). Therefore, considering the limited study, more research is needed to understand TFSV and its impact on the mental health of TFSV victims and their day-to-day lives. The purpose of this study was to describe victims’ responses to TFSV and to understand how TFSV influenced their lives and mental health. These findings intend to inform health care and policy concerning TFSV.

## Method

This is a qualitative study of a Korean website, which is entirely anonymous and was accessed publicly in May 2021. The website was opened in 2017 as a “Digital Sexual Crime Out” and began with an anonymous operator. The website is easily accessible through Korea's largest search engine. The purpose of the website is to share victims’ situations and information and allows users to respond together to the crime. The website has a bulletin board on which women who suffer from TFSV are free to post messages asking for help and voicing their concerns. Due to users’ sensitivity to information exposure, the website does not provide their personal information or the number of users on the website.

At the time of access in May 2021, there were 255 postings on the bulletin board. The bulletin board was intended for victims to share their concerns, but because some users wanted to form a social movement, several social news sources and advertisements were added. After a primary screening, postings unrelated to the bulletin board, such as news articles and advertisements, were excluded. A total of 54 posts that were all relevant to TFSV were selected. All data was extracted from the website and translated into English.

The data analysis for the identification of common themes followed the six-phase approach of Braun and Clarke (2012). This approach includes (1) familiarizing oneself with the data, (2) generating initial codes, (3) searching for themes, (4) reviewing potential themes, (5) defining and naming themes, and (6) producing the report. To improve the validity of results, the data was analyzed by authors 1 and 2, while author 3 supervised all the analytic processes. The posts and related conversations were then converted to 54 documents and uploaded to Atlas.ti for further analysis. The analysis followed a categorizing approach and systematic coding was used to explore large volumes of textual information without interference to determine trends, patterns, frequency, relationships, structures, and communication discourses. A preliminary codebook was developed by author 1. Authors 1 and 2 coded all of data separately using Atlas.ti version 9. The codebook was adjusted as needed, and if there were any discrepancies, discussion among all authors continued until agreement. This study was submitted to the Institutional Review Board at the University of Texas at Austin for review and deemed exempt.

## Results

### General Description of the Data

A total of 54 posts on the bulletin board were relevant to TFSV. The shortest post was one sentence with 8 words, excluding the title, while the longest post was 584 words. The numbers of comments on the posts ranged from 0 to 11, with an average of 2.54 comments per post. The post that received the most comments was (#63), about the victim's hopelessness. Although she had made several efforts to punish the perpetrator and delete the sexual video that had been distributed against her will, she just wanted to disappear. She said, “It's an infinite repetition. I want to be born again. No, I don’t want to be reborn. I hope I disappear without pain.” Five comments from other victims expressed sympathy with her pain, like, “I'm writing comments because it sounds like my story. I want to die, but I don't know how, so I'm just spending my days” and “The writer is in the same situation as me. My identity has also been exposed and uploaded to illegal pornographic websites every day. There are more victims besides me. I don't think it's a life.”

### Five Dimensions of TFSV

Following the classification of TFSV by Henry and Powell (2018), the types of damage mentioned in each post were classified. Some were classified as duplicates because the damage they described was seen as an area of overlap, while 14 posts did not reveal the type of TFSV. The most common type of victimization expressed on this website was the fourth dimension, image-based sexual exploitation, with 36 posts (Table 1).

### Relationship with Perpetrator

**Figure 1.**
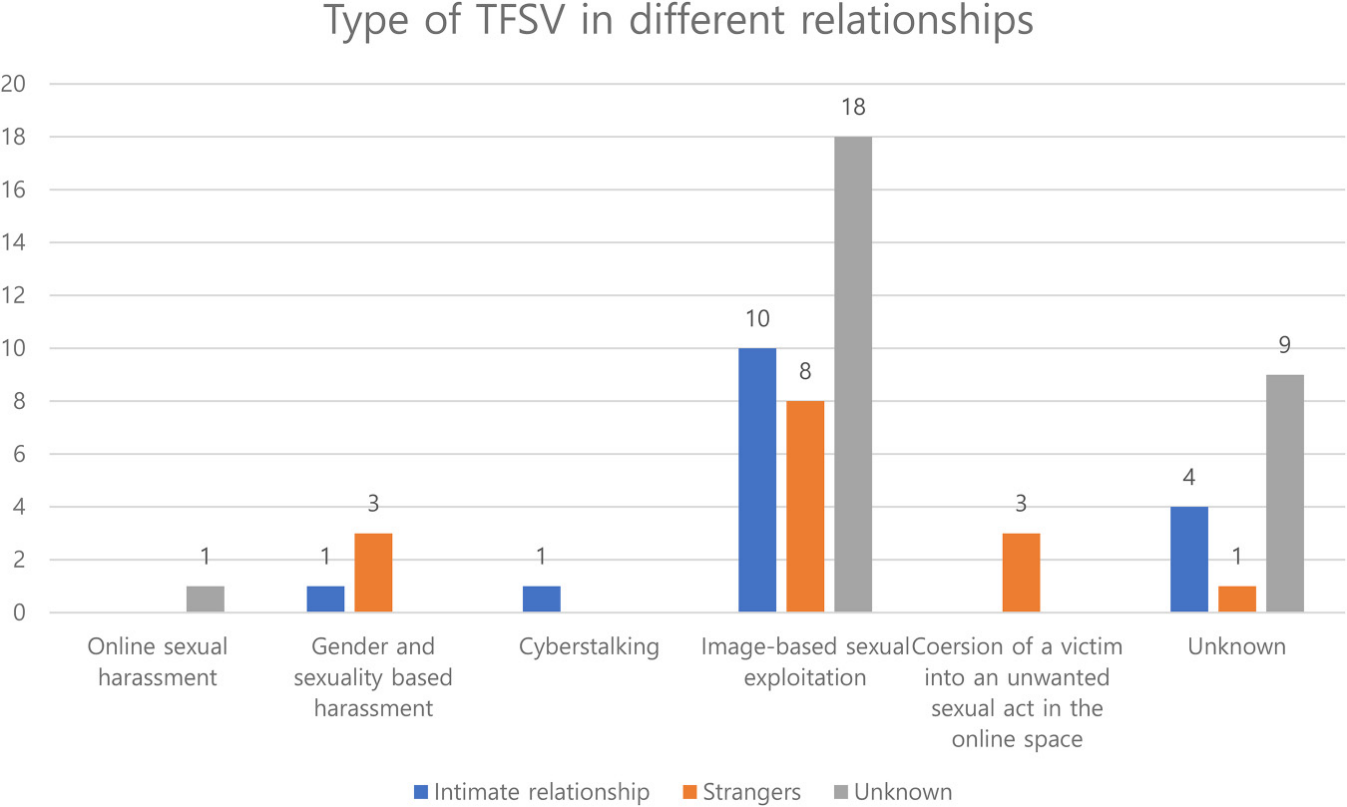
Type of TFSV in different relationships.

Among the 54 posts, 26 posts described the relationship between the victim and the perpetrator. Fourteen (53.85%) of these 26 posts, described TFSV that was perpetrated in an intimate relationship (e.g., current boyfriend, ex-boyfriend), while 12 posts (46.15%) described TFSV perpetrated by strangers. In the intimate relationships, unwanted distribution of images obtained by hidden camera were prevalent, including 6 of 14 cases. As for those perpetrated by strangers, fake composite photographs on adult sites were included. Compared to victimization by strangers, victims of intimate partners often resigned from their jobs, had difficulties in dealing with men, and felt impulses to harm the perpetrators (Figure 1).

### Victims’ Efforts to Resolve This Issue

Many victims started to screen adult websites when they noticed that their intimate pictures or videos had been distributed. If they found their contents in the online space, they contacted the website operators directly and asked them to delete the pictures or videos. Some of the victims used a company that helps to delete distributed contents by paying money. “At first, I paid $8,000 for the removal fee” (#32, reply 1). “A year ago, I spent $ 3,000 to delete the video” (#49). Deletion companies were created to erase traces of such posts on the Internet, a new industry in South Korea. Their customers request help because it is difficult to erase all the traces on their own after unwanted personal information has been distributed. This service is offered one time or on an ongoing basis, according to the customers’ requests. However, after it became known that several deletion companies also worked with illegal adult sites to generate more TFSV victims, deletion services were offered at the government level as far as TFSV is concerned (Bosco, 2022). Nonetheless, once the intimate contents were uploaded to the online space, the contents would come up again and again. They felt powerless: “I found out after I got a call from my friend. My video was uploaded on the website, and I first asked my friend to help me erase it, so I deleted it. Since then, I've been trying to erase one or two things a day, but even when erase one every day … 100 or 200 more appear. What should I do?” (#54).

Regarding the relationships between victims and the perpetrators, half of the victims of strangers tried to take action such as reporting the issue to the police or directly contacting the creators of the illegal website. Others, those who were victimized by intimate partners, discussed seeking help in three posts. Victims of intimate partners tried to solve the problem by reporting it to police, while those victimized by strangers primarily tried to directly contact the adult website and paid about a thousand dollars to a deletion company. On the other hand, the victims who could identify their perpetrators often contacted the police and punished the perpetrators, but if they could not, police officers told them it would be difficult to punish the perpetrators. “Every day is still hellish, and it's so hard. I sued, but the police said I can’t catch the disseminators because I can’t identify them” (#20, reply 1). “I filed a complaint with the police, but I heard, ‘We can't catch the criminal, it will take a long time.’ And finally, I couldn't catch him” (#41).

A few of the victims mentioned that they had tried to receive counseling but said that it was not satisfying. “I have gone to a counseling center with courage, but I just wondered why I was there. It's hard” (#36, reply 1). One victim shared that a therapist had made her upset. According to the post (#14), the therapist mentioned that people viewing the content might not recognize her face, name, school, or job, and the victims continued to say things like, “What the counselors say is not comforting to me. Should I undergo plastic surgery? Or should I change my name or go back to school? I want to die even though I'm not at fault. I hope that there is a solution. I just wrote my thoughts. I've been living only at home for a year.” Another victim in one post stated she is taking psychiatric medication (#63, reply 2).

### How TFSV Influences Victims’ Day-to-Day Lives

Several posts mentioned victims’ broken relationships with friends and impact on people close to them. In addition, some people stated that they had to resign from their jobs because they worried their coworkers might find out. Those who end their social relationships usually stay in their houses or even in their rooms. Furthermore, if victims’ day-to-day lives were impacted, their family members were also undergoing vicarious trauma or pains. “I've been living only at home for a year” (#14). “My sister stays in most of the rooms as if she had died, she locked the door and disconnected with other family members” (#52). Like the victims’ own suffering, their family members also have difficulties in dealing with this problem. “I'm sorry that my family is the only people whom I can tell this. My family is suffering from my stress” (#48). “My strong-looking mother tried to kill herself after that (daughter's suicide), and now she can't work [because of] depression and just stay[s] in her room. I am at a loss how to resolve my sister's injustice” (#52).

### Nature of Harm Experienced

Among the 54 posts, 15 posts mentioned victims’ suicidal ideations. Post #31 mentioned the victim's previous suicidal attempt: “Because of that trauma, I tried to kill myself and cut myself a lot.” And #52 mentioned her sister's (victim) loss by suicide. “My sister eventually tried to kill herself in February 2016 and died in a hospital after holding out for two years.” One victim felt hopeless because she thought it would be impossible to punish the perpetrators: “I've [already] sued, but I can't figure out where the distributors are. When I heard it, I tried to die by suicide” (#22, reply 5). One post mentioned the reason behind her suicidal ideation as shame: “I really want to die of shame” (#41, reply 1).

Twelve posts expressed victims’ anxiety regardless of whether or not they found their intimate videos or pictures in the online space. Those who did not find videos online always looked online because they knew or assumed that videos had been recorded or existed in their partners’ possession. As shown in #41— “I didn't find the video, but I feel like people around me are recognizing it. I saw men laughing at me.”—some victims felt anxious after breaking up with their partners just because they had filmed intimate videos. The online space is too large to find everything, and the victims felt this anxiety. Eleven posts mentioned victims’ fear of the world rather than their perpetrators. They did not express their feelings in detail, but it could be that their fear was related to the threat of change in their ordinary lives. When they wasted their time screening or deleting their intimate videos or pictures, they stated their anger and frustration: “I have to work but it's too unfair to do everything alone [No one can help me, so I have to solve it by myself]” (#41, reply 2). Once they felt frustrated, they usually asked advice on the website. They commonly asked, “How do I live?” (#2, 5, 6, 12, and 49). Five posts concerned victims’ sleep disturbance and depression, but only one post mentioned that the victim took psychiatric medication. “Even though I took psychiatric medicines, I feel like I can't sleep, I can't eat but I cry, and I feel heart-broken every day” (#48). Last, three posts expressed victims’ inability to focus on their work and lives.

### What They Need

In the posts, victims sought help in finding information, political or law enforcement, emotional support, and financial support. Among 36 of victims’ stated needs, 9 sought advice on finding or monitoring their intimate videos or pictures in the online space. Six posts involved victims’ wanting to know how they could delete their content on the adult site. “What should I do to sites that don’t delete what I requested?” (#40), “Does anyone know how many days it usually takes to delete it?” (#59). People who had a tough time deleting the content by themselves asked for information about the deletion company. However, this deletion company is not an authorized company, so victims doubted its authenticity. “Is it true that the digital deletion company is linked to adult sites?” (#44, reply 2). “Can I believe that he (deletion company) told me to sign the contract first and pay in advance if I want to erase it?” (#45).

Among 23 of victims’ political or law information needs, 11 posts asked how to report the issue to the police and what evidence would be needed to punish the perpetrators. Eight posts argued that a macro level of power, such as governmental or national, should be involved to solve this issue by increasing perpetrators’ criminal sentences. However, several posts were skeptical of these expectations: “I wish the country would stop it by law, but I don't think it's going to be too soon” (#41, reply 2). “It's horrifying to see the country's complacency” (#41, reply 6). On the other hand, 11 posts expressed a desire for emotional support and hope in finding a support group. They mentioned that there was no place to talk about the issue. “I think it's getting worse and worse because there's no place to talk about it” (#36, reply 1). “I'm afraid I'm going to explode because I can't speak anywhere else, so [if] I found a place like this I'd like to have a consultation” (#55).

## Discussion

Online based crime has been the topic of much debate since the 1990s, but there was little interest in TFSV (Henry & Powell, 2015b). The aim of this study was to explore victims’ responses and their efforts in the situation of TFSV; understand victims’ suffering in their day-to-day lives, especially from a mental health perspective; and discover their needs to detect or prevent risky behavior that may lead to health problems.

In a meta-analysis and systematic review of 19 articles, the prevalence of victimization regarding TFSV results showed that from 7.2 to 17.6% of people have experienced TFSV (Patel & Roesch, 2020). Reed et al. (2019) supported that 68.6% of participants reported experiencing at least one form of TFSV. There was considerable redundancy, which means that participants who reported one type of TFSV often reported experiencing other types as well. However, we cannot find studies conducted in Asian countries regarding TFSV prevalence. To accumulate scientific knowledge regarding TFSV, studies using the same classification are needed.

Pashang et al. (2019), found among 25 victims of TFSV, that 21 knew the perpetrators as friends, classmates, and ex-boyfriends. They also found that, contrary to the general perceptions, young girls actively engaged in TFSV, like their male counterparts, against other women. Reed et al. (2019), found that perpetrators of TFSV identified as male, but victims classified their relationships as “did not know” (41.8%), “met but did not know that well” (31.6%), “knew but did not date or have a relationship with” (31.6%), “were dating or in a relationship with” (26.6%), “were friends with” (24.1%), “hooked up with” (19.0%), or “liked or had a crush on” (12.7%). Due to limitations of this study, the relationships between perpetrators and victims are sometimes unclear. In the current study, 14 out of 26 posters (53.85%) said the perpetrator was an intimate partner, 12 posters (46.15%) mentioned they were strangers, and the relationship in the remaining 28 posts was unknown. Additionally, through the language they used (e.g., boyfriend, ex-boyfriend), it was assumed that all the perpetrators in intimate relationships were female. In order to compare results in other countries, more detailed study on relationships is needed.

The mental health impacts of TFSV are complex and long lasting, affecting young adult females’ personal and social lives (Pashang et al., 2019). Our study found that TFSV leaves permanent and public memories of shame, self-blame and stigmatization by known and unknown others. TFSV can lead victims to isolate themselves from friends, families, social places, employment, and education as well as other aspects of ordinary life. Because perpetrators can remain anonymous and are rarely convicted, victims fear recurring cyber exposure or its threat. Patel and Roesch (2020) conducted a qualitative analysis of nine articles to evaluate the relationship between TFSV victimization and mental health problems and found a significant impact on mental health including symptoms of anxiety, depression, and inability to cope. Additionally, an association between TFSV and poor health outcomes such as self-harm and suicidality was found. Snaychuk and O’Neill (2020) also found that victims of TFSV in the young adult population tended to have low self-esteem and high depressive symptoms. The current study found that victims reported anxiety, depression, fear, frustration, and suicidality. These findings indicate that TFSV should be recognized as a serious crime, and more work is needed to identify the health consequences associated with experiencing TFSV. The importance of interventions for TFSV victims’ physical and mental health must be emphasized.

Henry and Powell (2015a) argue that harmful digital communication is thought to be related to people's naïve attitudes rather than gender-based violence. They also argue that current legal and policy approaches do not capture the social and psychological damage inflicted on victims. Many posts in our study argued that progressive changes and enforced laws are needed to promote gender equality and prevent TFSV against women. On the other hand, Pashang et al. (2019), point out that victims need support groups with whom to share their feelings and that mental health experts need to be aware of the victims’ overall health and well-being. Since this study found that victims need support groups to share their negative feelings and to prepare further actions against TFSV, interventions including support group format are underscored.

Our study recommends enhanced public education and awareness of TFSV for society as well as health care providers to include knowledge concerning the mental health outcomes of TFSV in South Korea. Based on these results, conduct of further study using other research methodology is indicated. The current study findings can be used as a basis for legislative recommendations in South Korea.

Our study has several limitations. First, the study used a dataset from a public, anonymous website, and results do not reflect all aspect of TFSV in Korean society. Because the information of victims is not clear, more focused research concerning specific types of TFSV or specific ages is needed. Additionally, due to potential error related to memory recall, the content may be inaccurate or biased. However, due to the difficulty and sensitivity of assessing victims of TFSV, this study's analyzing of online communities demonstrates the genuine difficulties they confront in their ordinary lives. Further investigation is needed to explore other cultural phenomena influencing South Korea's TFSV for comparisons with circumstances in other countries.

## Conclusion

The current study explores the responses of victims of TFSV and their efforts, mental health concerns, and needs to detect or prevent risky behavior that may lead to health problems. These findings provide an empirical basis for future research aimed to prevent TFSV and protect victims’ physical and mental health in this country through increased access to mental health care.

**Table 1.** Frequency of TFSV Dimensions.

Dimensions	Frequency	Description
Online sexual harassment	1	One case (#62) was identified as online sexual harassment; In this case, someone uploaded the victim's personal information, including her photos, to Tumbler, and the victim suffered sexual harassment from strangers in the online space.
Gender- and sexuality-based harassment	4	Four cases were identified as fitting this dimension. Three of them (#4, 6, 13) involved fake composite photographs on adult sites. One case (#16) involved threats of video distribution by strangers.
Cyberstalking	1	One case (#3) involved cyberstalking. In this case, the victim's ex-boyfriend couldn't accept the breakup and kept contacting her. He threatened distribution of intimate photos and videos. She was concerned about how to handle this and asked if it would be better to sue him or meet with him again to make him erase the photos and videos.
Image-based sexual exploitation	36	Among these 36 cases, 8 cases concerned hidden cameras. In case #2, the victim found her video on a website. “Two years ago, I found a website with a video taken of me in the toilet.” The other victims found their pictures or videos in the perpetrators’ places. For example, in case #18, “I found several videos on his computer, and it made my life stumble; I couldn't even live. I saw a number of videos of him having sexual intercourse with women, including me, and I realized he was meeting with a lot of women at the same time, even when I was dating him. I don't even know when I was being shot. All the videos were secretly taken by him.” In case #25, “I woke up at home with my boyfriend, but I couldn't sleep, so I was looking at his cell phone. I went into the photo gallery, and I thought I was fainting. There were pictures from a hidden camera. They were taken on subways, at bus stops, and even in libraries and cafes.” In case #26, “My boyfriend tried to sneak a picture of me. We often cooked meals and watched movies together at my boyfriend's home. I caught him trying to do a hidden camera.”
Coercion of a victim into an unwanted sexual act in the online space	3	Three cases (#19, 28, 31) were identified as coercion. These cases seemed to be written by adolescents. In the posts, one victim said, “About four years ago, I was forced to share a video of my face in a chat room with about 30 people because of intimidation” (#28). Another said, “I was 12 years old at the time. Four years ago, because of the man's intimidation, I sent my videos and photos to him” (#19). In this case, the victim seems more anxious than victims of other cases even though she actively responded to solve this problem.
Unknown	14	
